# Supplementary material for: Lactic Acid Bacteria Fermented Cordyceps militaris (GRC-SC11) Suppresses IgE Mediated Mast Cell Activation and Type I Hypersensitive Allergic Murine Model
Source: Nutrients. 2021 Oct 28;13(11):3849. doi: 10.3390/nu13113849 (PMC8618942; doi:10.3390/nu13113849)
Supplement: Supplementary file 1 [file nutrients-13-03849-s001.zip › nutrients-1413350-supplementary.pdf]

nutrients

MDPI

Supplementary File

Supplement Table S1. Lactic acid bacteria strains used for fermenting GRC

Abbreviation	Strain	Source
GRC-SC11	Pediococcus pentosaceus SC11	Small octopus salted Seafood
GRC-ON89A	Pediococcus pentosaceus ON-89A	Onion
GRC-ON188	Pediococcus pentosaceus ON-188	Onion

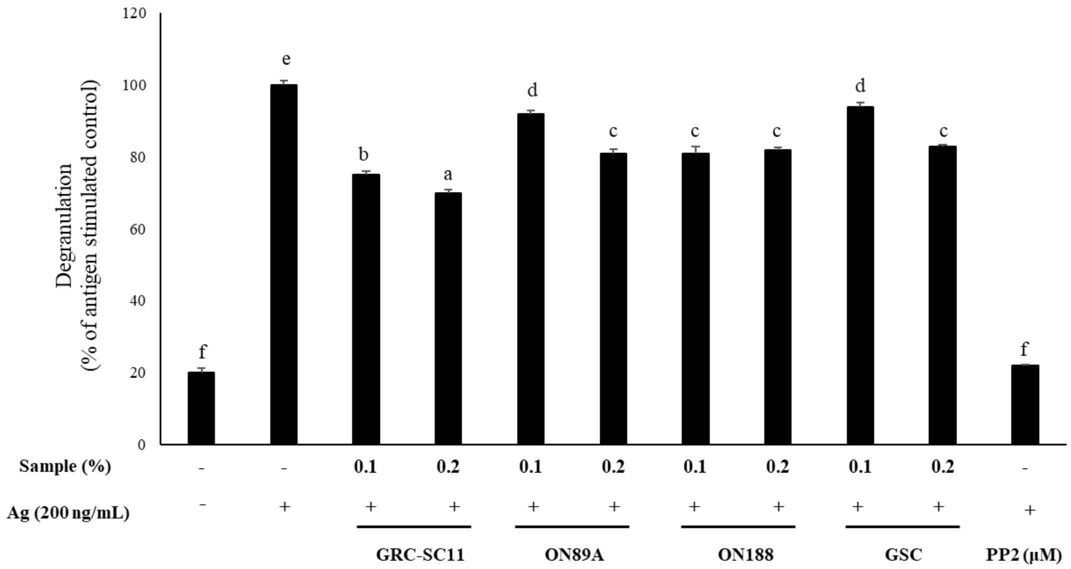

Supplement Figure S1. Effect of GRC fermented with lactic acid bacteria on β-hexosaminidase assay from RBL-2H3 stimulated with IgE/Ag. Data were analyzed by one-way ANOVA/Duncan's t-test ($p < 0.05$). Different letters indicate significant differences between groups.
